# Development and Validation of a High-Resolution Melting (HRM) Method for Differentiating Ovis and Equi Biovars of *Corynebacterium pseudotuberculosis*

**DOI:** 10.3390/vetsci13040372

**Published:** 2026-04-13

**Authors:** Jingpeng Zhang, Dingding Zhang, Jinxiu Jiang, Yusheng Lin, Chunhe Wan, Yongliang Che

**Affiliations:** 1 Institute of Animal Husbandry and Veterinary Medicine, Fujian Academy of Agricultural Sciences, Fuzhou 350013, China; qm870063543@163.com (J.Z.);; 2 College of Animal Science, Fujian Agriculture and Forestry University, Fuzhou 350002, China

**Keywords:** *Corynebacterium pseudotuberculosis* biovar ovis, *Corynebacterium pseudotuberculosis* biovar equi, *gyrA* gene, high-resolution melting

## Abstract

*C. pseudotuberculosis* is a Gram-positive pathogenic bacterium that infects various animals, causing diseases such as caseous lymphadenitis, leading to significant economic losses in the livestock industry and posing zoonotic risks. This study targeted the conserved *gyrA* gene fragment of this bacterium, designed specific primers, optimized the reaction system and conditions, and established a high-resolution melting curve (HRM) detection method with potential utility for preliminary molecular screening. Validation showed that this method exhibits strong specificity, producing specific amplification only for the target biovars. The melting temperatures (Tm values) for the two biovars were 86.16 ± 0.05 °C and 86.92 ± 0.05 °C, respectively, allowing clear differentiation. It demonstrated high sensitivity, with minimum detection limits of 28 copies/μL and 25 copies/μL for standard plasmids of the ovis and equi biovars, respectively. The method also showed good reproducibility, with intra- and inter-batch coefficients of variation both below 1.0%. Applied to 133 clinical nasal swab samples from goats in Fujian Province, the method detected a positivity rate of 19.5% and indicates that a biovar equi-like gene fragment was detected in goat nasal swabs from Fujian Province via molecular screening. The HRM method developed in this study is sensitive, specific, simple, and cost-effective, enabling rapid detection and biovar differentiation of *C. pseudotuberculosis*. It is suitable for large-scale clinical sample screening and provides an efficient technical approach for epidemiological monitoring and precise control of the disease.

## Simple Summary

*Corynebacterium pseudotuberculosis* has two biovars, ovis and equi, which cause severe diseases in livestock and pose zoonotic risks., rapid differentiation of these biovars is critical for disease control. This study developed a high-resolution melting detection method targeting the conserved DNA gyrase subunit A gene of the bacterium. Specific primers were designed and reaction conditions were optimized to accurately differentiate the two biovars. The method exhibited strong specificity (no cross-reaction with other pathogens), high sensitivity (detection limits of 28 and 25 copies/μL for ovis and equi biovars), and good reproducibility (intra- and inter-batch CV < 1.0%), with distinct melting temperatures for clear biovar distinction. Applied to 133 goat nasal swab samples from Fujian Province, it achieved a 19.5% positive rate. This high-resolution melting method is simple, cost-effective, and suitable for large-scale clinical screening, providing an efficient technical tool for epidemiological monitoring and precise control of *Corynebacterium pseudotuberculosis* infections in livestock.

## 1. Introduction

*Corynebacterium pseudotuberculosis* (*C. pseudotuberculosis*) is a Gram-positive, facultative intracellular parasitic bacterium belonging to the class Actinobacteria, order Mycobacteriales, family Corynebacteriaceae. It is a pathogen for various animals, including sheep, goats, horses, camels, and other wild herbivores [1,2,3], and occasionally causes chronic infectious diseases in humans [4]. This bacterium is widely distributed globally, causing significant economic losses to the livestock industry. Notably, this pathogen possesses significant zoonotic potential and is primarily an occupational zoonotic agent, posing potential threats to public health and safety [5,6,7].

Based on nitrate reductase activity, *C. pseudotuberculosis* is typically divided into two biovars: the equine biovar (biovar equi) and the ovine biovar (biovar ovis). The biovar ovis strains are primarily isolated from sheep and goats, exhibit negative nitrate reduction, and can cause caseous lymphadenitis (CLA) in sheep and goats [8]. CLA is a chronic infectious disease with three clinical manifestations: superficial, visceral, and mixed types [4], among the clinical forms of *C. pseudotuberculosis* infection, the superficial form is the most common. It leads to reduced meat, milk, and wool production in affected animals, causing significant economic losses in the global sheep and goat industries. For example, a seroprevalence study in goats in Espírito Santo, Brazil, revealed a high *C. pseudotuberculosis* infection rate of 34.5% [9]. In Korean native goats, the distribution of CLA and its association with gender and age have also been investigated (infection rate of 13.98–28.16%) [10]. A 15-year molecular characterization study in Switzerland demonstrated that biovar ovis strains are the primary pathogens responsible for caseous lymphadenitis in sheep and goats [11]. In southwestern China, the isolation and molecular characterization of *C. pseudotuberculosis* from external abscesses in goats have been reported, showing a high isolation rate (infection rate of 39.22%) [12]. These studies contribute to understanding the epidemiological features of CLA and provide a basis for disease control. The biovar equi strains exhibit positive nitrate reduction and are mainly isolated from horses, cattle, camels, and other animals, causing external abscesses, intra-abdominal or thoracic abscesses, and ulcerative lymphangitis [13,14]. Molecular epidemiological research in the USA found an increasing trend of *C. pseudotuberculosis* infections in horses (infection rate of 54%) [13]. In Mexico, biovar equi strains were first isolated from muscle abscesses in two horses. Researchers suggest that the pathogen’s infection rate in horses may be higher than previously expected [15].

The most significant route of infection for *C. pseudotuberculosis* is skin abrasions that become contaminated, leading to superficial caseous lymphadenitis [16]. Animals infected with *C. pseudotuberculosis* continuously shed live bacteria into the environment. Due to the unique cell wall structure of *C. pseudotuberculosis*, the bacteria can persist in the environment for extended periods, reports of survival ranging from 2 to 8 months in wool, hay, and soil [17], leading to repeated infections in farm animals [18], which affects their production performance and causes economic losses. Currently, treatment for *C. pseudotuberculosis* is often ineffective, commercial inactivated vaccines offer limited protection, and novel vaccines are still under development [19]. Therefore, rapid and accurate diagnosis combined with culling of infected animals are crucial for effective prevention and control. Traditionally, biochemical, serological, and molecular methods have been used for diagnosing *C. pseudotuberculosis* [20]. To improve diagnostic efficiency and accuracy, researchers have developed multiplex PCR detection methods capable of simultaneously detecting and distinguishing between the two biovars of *C. pseudotuberculosis* [21]. One study utilized hypervariable regions of the rpoB gene to develop a PCR-restriction analysis method for identifying *C. pseudotuberculosis* in sheep [22]. Additionally, MALDI Biotyper and the overall genome-related index (OGRI) analysis have been evaluated for optimizing the identification and biotype differentiation of *C. pseudotuberculosis* [20]. However, these methods are time-consuming, costly, and present other challenges.

High Resolution Melting (HRM) is a qPCR technique used for screening and detecting DNA mutation sites in target regions [23]. It is a post-PCR mutation detection method and explicitly states that the intercalating dye binds only to double-stranded DNA, producing distinct melting curves depending on GC content and sequence composition of the amplicon. Its principle involves saturating the PCR reaction with fluorescent dyes that do not affect amplification efficiency. After the PCR reaction is completed, a gradual heating process causes the target fragments to slowly denature and unwind, leading to a gradual decrease in fluorescent signals. The presence of mutation sites within the target fragments can result in subtle differences in denaturation, thereby affecting variations in fluorescent signals. This technology was invented by American scientists in 2003 and offers numerous advantages, including high sensitivity, speed, accuracy, and high throughput [24]. It has currently demonstrated strong discriminatory capabilities and advantages in the detection of bacteria, fungi, viruses, and parasites [25,26,27,28]. To date, this technique has not been applied to the detection of *C. pseudotuberculosis*. The *gyrA* gene encodes subunit A of bacterial DNA gyrase. Based on transcriptomic data of *C. pseudotuberculosis* under different stress conditions, Carvalho et al. identified the gyrA gene as one of the most stably expressed genes in *C. pseudotuberculosis*, making it suitable for standardization in RT-qPCR studies of this pathogen [29]. In this study, specific primers were designed based on the conserved regions of the *gyrA* gene of *C. pseudotuberculosis* to establish an HRM detection method for identifying and distinguishing between biovar ovis and equi strains of *C. pseudotuberculosis*. This aims to provide technical support for the differential diagnosis, prevention, and control of *C. pseudotuberculosis*.

## 2. Materials and Methods

### 2.1. Bacterial (Viral) Strains and Clinical Samples

The standard strains involved in this study include *C*. *pseudotuberculosis* biovar equi (CCUG43567), *Corynebacterium ulcerans* (*C. ulcerans*) DNA standard (BNCC364012), and *Staphylococcus aureus* (*S*. *aureus*, ATCC25923). The *C*. *pseudotuberculosis* biovar ovis strain FJ-PN, *Orf* virus (*ORFV*), *Pasteurella multocida* (*P. multocida*), *Mycoplasma ovipneumoniae* (*M. ovipneumoniae*), and *Mycoplasma mycoides subsp. capri* (*M. mycoides subsp. capri*) were isolated, identified, and preserved by our laboratory [30]. *Mycoplasma capricolum subsp. Capripneumoniae* (*M. capricolum subsp. Capripneumoniae*) was kindly provided by Dr. Chu Yuefeng from the Lanzhou Veterinary Research Institute. A total of 133 nasal swab samples from goats, collected between 2019 and 2025 from various goat farms in Fujian Province, were from clinically suspected CLA cases.

### 2.2. Main Reagents and Consumables

The fluorescent dye Syto9 was purchased from Thermofisher (Invitrogen, Thermo Fisher Scientific Cat#S34854, Waltham, MA, USA); PCR amplification reagent Premix Taq™ Hot Start Version (Cat#R028Q) was purchased from Takara Bio Inc.(Beijing, China); bacterial nucleic acid extraction kit EasyPure Viral/Bacteria DNA/RNA Kit (Cat#ER201), T-cloning vector kit pMD19-T Vector Cloning Kit (Cat#6013), gel extraction kit Quick Gel Extraction Kit (Cat#EG101), and Plasmid MiniPrep Kit (Cat#EM101) were all purchased from TransGen Biotech (Beijing, China); fluorescent quantitative eight-tube strips (PCR-0208-C) were purchased from Axygen Scientific Inc. (Union City, CA, USA); other conventional chemical reagents and consumables were purchased from Sangon Biotech (Shanghai, China).

### 2.3. Cloning of Target Genes

#### 2.3.1. Conventional PCR Primer Design

For cloning of the target gene, specific primers need to be designed based on the target sequence. The conserved sequences of the *gyrA* gene from *C. pseudotuberculosis* biovar ovis and biovar equi strains registered on Genbank were referenced in this study. A pair of specific primers was designed using Primer Premier 5.0 software. The primers were named CP-GyrAF and CP-GyrAR, with sequences 5′-CTTGGCGTGGTTACCTTCAAGT-3′ and 5′-GCTCCTTCAGGCTCAATGTTC-3′, respectively. The primers were synthesized by Boshang Biotechnology Co., Ltd. (Shanghai, China), with an expected amplicon length of 320 bp.

#### 2.3.2. PCR Amplification of the Target Sequence

*C. pseudotuberculosis* biovar ovis and *C. pseudotuberculosis* biovar equi strains were revived on sheep blood agar plates, single colonies were picked and inoculated into TSB supplemented with 10% Fetal Bovine Serum (FBS) for cultivation. Sterile PBS was added to the *C. pseudotuberculosis* biovar ovis and biovar equi cultures at a volume ratio of 1:3, followed by thorough mixing and three consecutive freeze–thaw cycles. The mixtures were centrifuged at 4000 rpm for 20 min, and the supernatants were discarded subsequently. DNA was extracted from the bacterial cultures using the EasyPure Viral/Bacteria DNA/RNA Kit in accordance with the manufacturer’s instructions. The target gene was amplified from the extracted DNA using the specific primers (CP-GyrAF and CP-GyrAR) which were designed in Section 2.3.1. The 50 μL PCR reaction system was prepared as follows: 25 μL of 2 × TransTaq-T PCR SuperMix, 1 μL each of 10 μmol/L specific forward/reverse primers, 1 μL of DNA sample, and Nuclease-free Water added to a final volume of 50 μL. Amplification was performed with the following thermal cycling conditions: pre-denaturation at 94 °C for 5 min; 40 cycles of 94 °C denaturation for 30 s, 55 °C annealing for 30 s, and 72 °C extension for 60 s; followed by a final extension at 72 °C for 10 min. The PCR products were analyzed by 1.0% agarose gel electrophoresis. Target fragments were excised and purified using a gel extraction kit, then cloned into the pEASY-T1 Simple Cloning Kit vector. Positive recombinant plasmids were screened via standard protocols and submitted to Boshang Biotechnology Co., Ltd. for Sanger sequencing.

#### 2.3.3. Sequence Alignment Analysis

Sequencing results were validated via BLAST (Basic Local Alignment Search Tool, https://blast.ncbi.nlm.nih.gov/Blast.cgi, accessed on 5 December 2025) analysis against the NCBI database. A total of 167 *C. pseudotuberculosis* strains, including 90 isolates of *C. pseudotuberculosis* biovar ovis and 77 isolates of *C. pseudotuberculosis* biovar equi, were deposited in the NCBI database, covering major global epidemic regions (South and North America, Europe, Middle East, Asia, Africa, Oceania). For subsequent gyrA gene analysis, strains were selected from geographically diverse regions, and isolates with ambiguous biotype classification or incomplete host annotation data were excluded. The validated *gyrA* gene sequences were aligned against reference *C. pseudotuberculosis* strains in the NCBI database using MegAlign 7.10 to analyze nucleotide homology. Phylogenetic relationships among the strains were determined using MEGA7.0 (version 7.0.14). A neighbor-joining (NJ) phylogenetic tree was constructed with 1000 bootstrap replications to assess the reliability of branching topology.

### 2.4. Establishment of HRM Detection Method

#### 2.4.1. Primer Design for HRM

**Figure 1:**
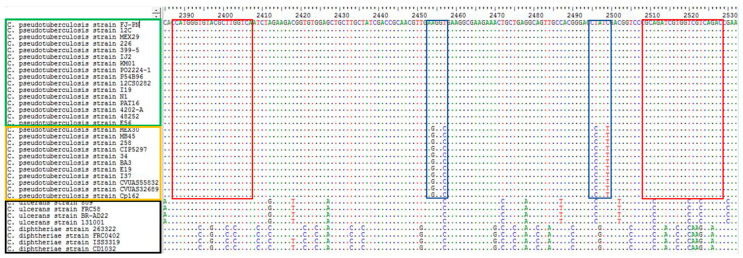
Multiple sequence alignment was performed using BioEdit software (Version 7.2.6) with sequences retrieved from NCBI. Red boxes denote complete complementarity between primers and *C. pseudotuberculosis* biovar ovis and biovar equi; blue boxes indicate base mismatches in *C. pseudotuberculosis* biovar ovis and biovar equi. Green boxes represent strains of *C. pseudotuberculosis* biovar ovis, yellow boxes represent strains of *C. pseudotuberculosis* biovar equi, and black boxes represent strains of other *Corynebacterium* species. Base colors: red = T, blue = C, green = A, black = G. Identical bases are indicated by color-coded dots.

Based on the characteristics of the *gyrA* gene analyzed in Section 2.3.3, specific primers for HRM were designed using Oligo (v7.37). The *C. pseudotuberculosis* biovar ovis (CP011474) and *C. pseudotuberculosis* biovar equi (CP017291) were used for primer design, while the strain FJ-PN isolated in our laboratory and *C. pseudotuberculosis* biovar equi (CCUG43567) was used for fragment amplification. The primer sequences were 5′-CATGGGTGTACGCTTGGTCA-3′ and 5′-GTCTGACGACCACGATCTGC-3′, which were designated CP-GR2F and CP-GR2R, respectively, with an expected fragment size of 140 bp (Figure 1). Figure 1 shows the multiple sequence alignment used for primer design, ensuring specificity. The primers were synthesized by Boshang Biotechnology Co., Ltd.

#### 2.4.2. Reaction Condition Optimization

The plasmids containing the *gyrA* gene of *C. pseudotuberculosis* biovar ovis and *C. pseudotuberculosis* biovar equi, which were sequenced and identified in Section 2.3.3 (designated as PMD19T-GryAO and PMD19T-GryAE), were used as the positive standards in this study. After linearization of the plasmid with the restriction endonuclease (BamH I), the concentrations of the plasmids were measured using a NanoDrop spectrophotometer (Waltham, MA, USA), and the measured values were converted into copy numbers (2.8 × 10^10^ copies/μL and 2.5 × 10^10^ copies/μL, respectively). Ten-fold serial dilutions of the plasmid standards were prepared for subsequent experimental use.

A 20 μL HRM reaction system was prepared. Reaction conditions were optimized using the Roche LightCycler 96 real-time fluorescent quantitative PCR instrument (Mannheim, Germany), with different final primer concentrations (200, 400, 600, 800, and 1000 nM) and annealing/extension temperatures (54, 56, 58, 60, and 62 °C) with a 15 s duration. Following the completion of amplification cycles, optimal reaction conditions were selected based on the amplification curves and melting curves generated under the software-recommended parameters.

#### 2.4.3. Establishment of the Standard Curve

Six dilution levels of *C. pseudotuberculosis* biovar ovis and biovar equi standard plasmids, at concentrations ranging from 2.8 × 10^6^ to 2.8 × 10^1^ copies/μL and 2.5 × 10^6^ to 3.6 × 10^1^ copies/μL, respectively, were selected as templates for HRM reactions. Amplification was performed using optimized reaction conditions to obtain amplification curves. The common logarithm of the initial copy number of the standard (log quantity) was used as the abscissa, and the cycle threshold (Ct value) as the ordinate to plot the standard linear regression equation (standard curve) established in this study for the HRM reaction.

#### 2.4.4. Specificity Test

The optimized HRM reaction conditions were used to detect *C. pseudotuberculosis* biovar ovis and biovar equi, as well as common pathogens in sheep such as *Orf* virus (*ORFV*), *Pasteurella multocida* (*P. multocida*), *Mycoplasma ovipneumoniae* (*M. ovipneumoniae*), *Mycoplasma mycoides subsp. capri* (*M. mycoides subsp. capri*) and *Mycoplasma capricolum subsp. capripneumoniae* (*M. capricolum subsp. capripneumoniae*), to evaluate the specificity of the established method.

#### 2.4.5. Sensitivity Test

Serially diluted standard plasmids of *C. pseudotuberculosis* biovar ovis and biovar equi (with concentrations ranging from 2.8 × 10^2^–2.8 × 10^0^ copies/μL to 2.5 × 10^2^–2.5 × 10^0^ copies/μL) were used as templates for HRM reactions. Reactions were conducted under optimized conditions to determine the minimum detection limit.

#### 2.4.6. Repeatability Test

The established HRM method was used to detect the standard plasmid of *C. pseudotuberculosis* biovar ovis (with concentrations of 2.8 × 10^2^ copies/μL, 2.8 × 10^4^ copies/μL, 2.8 × 10^6^ copies/μL) as well as the standard plasmid of *C. pseudotuberculosis* biovar equi (with concentrations of 2.5 × 10^2^ copies/μL, 2.5 × 10^4^ copies/μL, 2.5 × 10^6^ copies/μL). Each standard concentration was tested in triplicate to calculate the intra-group coefficient of variation. The above standards were aliquoted and stored at −20 °C, and taken out every 7 days for detection using the established HRM method. A total of 3 detections were conducted to calculate the inter-group coefficient of variation.

### 2.5. Clinical Sample Detection

The nylon flocked swab was gently inserted into one nostril along the floor of the inferior nasal meatus to a depth of approximately 3 cm, rotated 3–5 times, and then slowly withdrawn. The swab was immediately immersed in preservation solution, the swab shaft was broken, and the tube cap was tightly secured; care was taken to ensure that the swab tip did not contact any non-sampling surfaces. After collection, the swabs were placed into sterile tubes, stored at 4 °C during transportation, and processed within 48 h. For 133 nasal swabs (Nylon flocked swabs), sterile PBS (volume ratio 1:3) was added and mixed thoroughly. After three freeze–thaw cycles, DNA was extracted using a bacterial genomic DNA/RNA extraction kit according to the manufacturer’s instructions. All samples were tested using both the in-house established HRM method and the TaqMan-qPCR method [31], the latter of which exhibits superior specificity and has become a standard for microbial identification. and the concordance rate between the two detection approaches was verified. The positive control was the genome DNA of *C. pseudotuberculosis* biovar ovi and biovar equi, Sterile double-distilled water (ddH_2_O) was used as the no-template negative control in each PCR/HRM run to monitor for reagent contamination or non-specific amplification. All positive nucleic acid test nasal swabs were inoculated onto blood agar plates (containing 5% sheep blood) for bacterial culture at 37 °C for 48 h, and the isolation and identification was conducted in accordance with the protocol described in reference [30].

## 3. Results and Analysis

### 3.1. Amplification Results of the Target Gene

**Figure 2:**
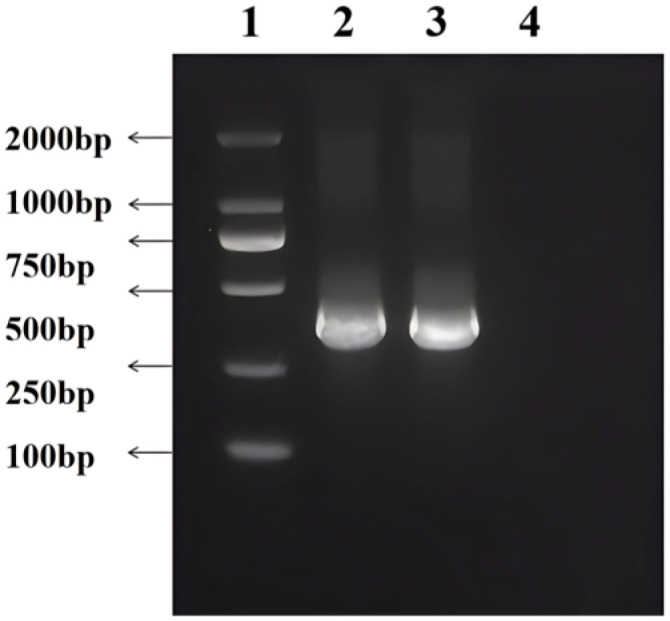
Amplification results of *gyrA* gene of *C. pseudotuberculosis* biovar ovis and *C. pseudotuberculosis* biovar equi. 1. DNA marker (DL2000). 2. Amplification product of *gyrA* gene of *C. pseudotuberculosis* biovar ovis (320 bp). 3. Amplification product of *gyrA* gene of *C. pseudotuberculosis* biovar equi (320 bp). 4. Blank control.

The *gyrA* genes of *C. pseudotuberculosis* biovar ovis and biovar equi were amplified by PCR, and a target band of 320 bp was observed after agarose gel electrophoresis (Figure 2). The target fragment was gel-purified, cloned, and sequenced. Sequence alignment via BLAST showed that the sequences were consistent with the published *gyrA* gene sequences of *C. pseudotuberculosis* biovar ovis and *C. pseudotuberculosis* biovar equi.

### 3.2. Nucleotide Homology Comparison of the gyrA Gene

**Figure 3:**
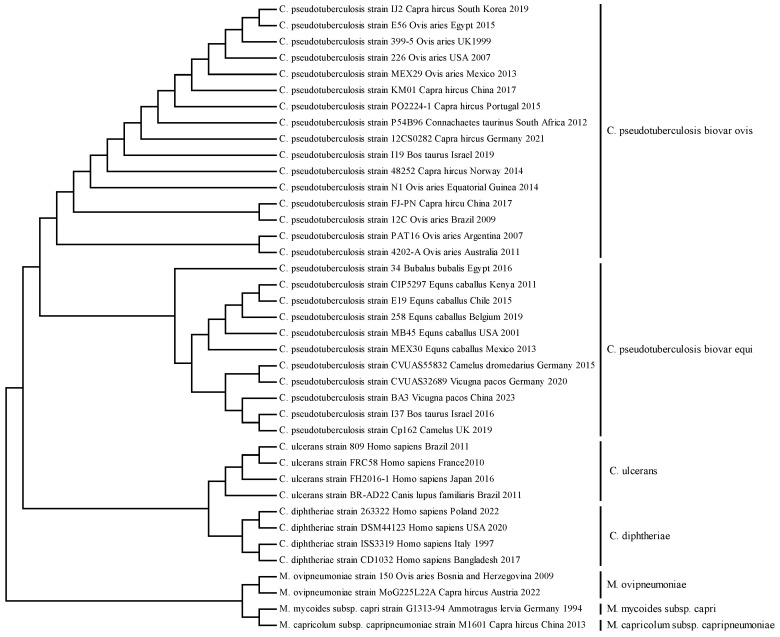
Phylogenetic analysis was performed based on the gyrA gene of *Corynebacterium pseudotuberculosis*. A neighbor-joining (NJ) phylogenetic tree was constructed with 1000 bootstrap replicates. *C. pseudotuberculosis* strains were clustered into two distinct clades corresponding to biovar ovis and biovar equi, respectively. The phylogenetic relationships confirmed the genetic distinctiveness of each species and biovar, with clear separation between the two biovars of *C. pseudotuberculosis*. Meanwhile, *C. ulcerans* and *C. diphtheriae* each formed independent evolutionary lineages. The three mycoplasma species also clustered into distinct and well-separated phylogenetic clades. Detailed information on the strains used for phylogenetic tree construction is shown in Table S2.

The nucleotide homology comparison results showed that the FJ-PN strain shared 99.92–99.96% nucleotide homology with the *gyrA* gene of other *C. pseudotuberculosis* biovar ovis strains and 99.10–99.18% with *C. pseudotuberculosis* biovar equi strains in GenBank. Compared to members of the genus *Corynebacterium*, the homology is relatively higher with *C*. *ulcerans* (88.58–89.16%) and *C*. *diphtheriae* (80.10–80.31%), while the nucleotide homology with other members is below 80.00%. Phylogenetic tree shows that all biovar ovis strains cluster into a single, highly supported independent clade, and all biovar equi strains form another distinct clade. Strains from different geographic regions (South and North America, Europe, Middle East, Asia, Africa, Oceania) are uniformly clustered within the corresponding biovar clades without intermixing. The phylogenetic tree was shown in Figure 3. Moreover, we have conducted a full sequence alignment of the 140 bp *gyrA* target fragment of all 167 strains, and the results confirm that the four SNPs are strictly conserved as biovar-specific markers in all published global lineages: all biovar ovis strains (90) show identical base types at these four SNP loci, and all biovar equi strains (77) have consistent characteristic bases at the corresponding loci. A total of 4 nucleotide differences between the two biovars were identified at positions 2454, 2457, 2495, and 2499 of the *gyrA* gene, respectively. In the *gyrA* gene of biovar ovis, the nucleotide sequences are A, T, T, C, whereas in biovar equi they become G, C, C, T. detailed data are provided in Supplementary Materials (Table S1).

### 3.3. Optimization of HRM Reaction Conditions

The optimized HRM experimental conditions are as follows: the optimized primer concentration is 0.6 μmol/L, the entire reaction system contains 1× Taq pre-mix, 1 μmol/L Syto9 fluorescent dye, 1 μL template, and double-distilled water (ddH_2_O) is added to a final volume of 20 μL. The optimal annealing temperature was determined to be 58 °C; therefore, the reaction conditions were set as: 95 °C pre-denaturation for 2 min, followed by 40 cycles, each cycle including 95 °C denaturation for 15 s, 58 °C annealing for 5 s, and 72 °C extension for 10 s, totaling 20 s. The HRM analysis procedure is as follows: 95 °C for 1 min, 45 °C for 1 min, 50 °C for 10 s, then heating to 90 °C at a rate of 0.3 °C per second.

### 3.4. Establishment of Standard Curve and Amplification Efficiency Analysis

**Figure 4:**
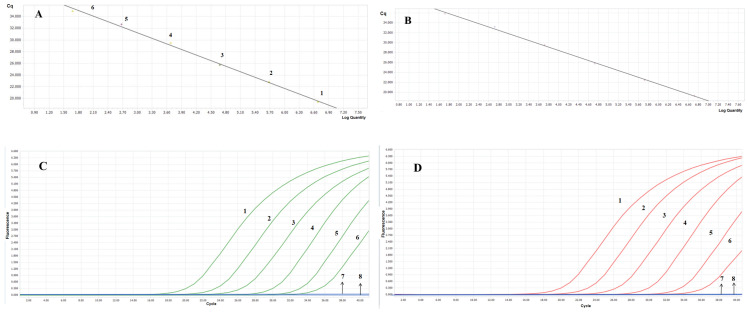
Standard curve and amplification curve of *C. pseudotuberculosis* biovar ovis and biovar equi. (**A**) Standard curve of *C. pseudotuberculosis* biovar ovis performed in a linear graph with R^2^ = 1.0 and a slope of −3.1803. (**B**) Standard curve of *C. pseudotuberculosis* biovar equi performed in a linear graph with R^2^ = 1.0 and a slope of −3.3857. (**C**) Amplification curve of the HRM method for *C. pseudotuberculosis* biovar ovis (green curve). (**D**) Amplification curve of *C. pseudotuberculosis* biovar equi (red curve). The numbers (1–6) correspond to 10^6^ to 10^1^ copies/μL of the standard plasmid dilutions. Arrows indicate samples with no amplification curves. Number 7 represents the 10^0^ copies/μL dilution and number 8 denotes ddH_2_O.

The standard gene plasmids of *C. pseudotuberculosis* biovar ovis and *C. pseudotuberculosis* biovar equi (PMD19T-GryAO, PMD19T-GryAE) were subjected to 10-fold serial dilution to achieve final concentrations of 2.8 × 10^1^–2.8 × 10^6^ copies/μL and 2.5 × 10^1^–2.5 × 10^6^ copies/μL, respectively, to construct standard curves. Linear regression analysis revealed that the HRM method for detecting *C. pseudotuberculosis* biovar ovis had a standard curve that showed a correlation coefficient (R^2^ = 1.00), a slope of −3.1803, and a Y-axis intercept of 40.77. The HRM method for detecting *C. pseudotuberculosis* biovar equi showed (R^2^ = 1.00), a slope of −3.3857, and a Y-axis intercept of 41.94. According to the formula E = 10^(−1/slope)^ − 1, the amplification efficiencies were calculated as PMD19T-GryAO (E = 106%) and PMD19T-GryAE (E = 97.4%), indicating that the reaction system had good amplification efficiency (Figure 4).

### 3.5. Specificity Validation

**Figure 5:**
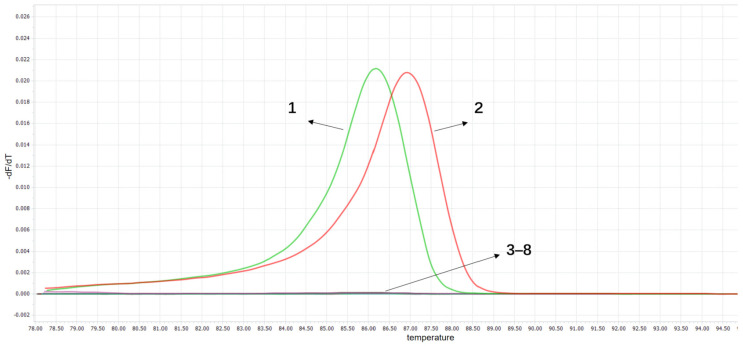
The specificity test for the HRM method. 1. *C. pseudotuberculosis* biovar ovis; 2. *C. pseudotuberculosis* biovar equi. 3–8: *P. multocida*, *ORFV*, *M. ovipneumoniae*, *M. mycoides subsp. Capri*, *M. capricolum subsp. Capripneumoniae*, ddH_2_O.

The HRM detection method established in this experiment was used to conduct specificity testing on *C. pseudotuberculosis* and common ovine pathogens (*P. multocida*, *ORFV*, *M. ovipneumoniae*, *M. mycoides subsp. capri*, *M. capricolum subsp. capripneumoniae*). The results showed that only *C. pseudotuberculosis* biovar ovis and *C. pseudotuberculosis* biovar equi samples exhibited melting curves, while no fluorescence signals were detected in other pathogens (Figure 5). Tm values analysis revealed that *C. pseudotuberculosis* biovar ovis formed a single melting peak with Tm value at 86.16 ± 0.05 °C, and *C. pseudotuberculosis* biovar equi formed a single melting peak with Tm value at 86.92 ± 0.05 °C. The Tm value difference between the two biovars was 0.76 ± 0.03 °C, allowing effective differentiation between them. No fluorescence signals were observed in other pathogens. The melting curves indicated strong specificity of the reaction.

### 3.6. Sensitivity Validation

**Figure 6:**
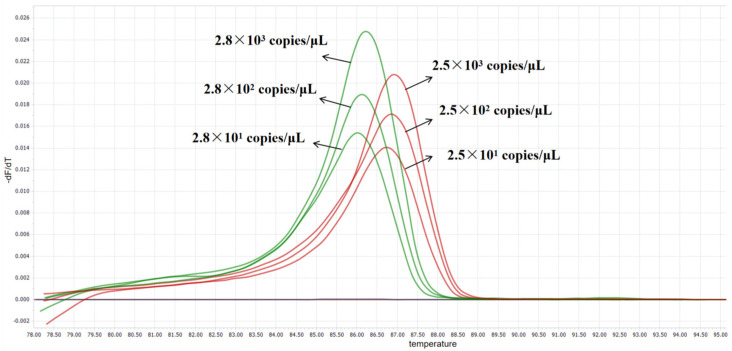
Sensitivity validation of the HRM assay: The green curve corresponds to *C. pseudotuberculosis* biovar ovis, whereas the red curve denotes *C. pseudotuberculosis* biovar equi. Both melting curves exhibited distinct peaks within the low-concentration range, with a mean Tm value difference of 0.76 ± 0.03 °C between the two amplicons.

Using the HRM method established in this study, a sensitivity test was conducted on 10-fold serially diluted standard plasmids. The results are shown in Figure 6. The minimum detection limit was 28 copies/μL (2.8 × 10^1^ copies/μL) for *C. pseudotuberculosis* biovar ovis, and 25 copies/μL (2.5 × 10^1^ copies/μL) for *C. pseudotuberculosis* biovar equi, indicating that the established HRM method has high sensitivity.

### 3.7. Repeatability Validation

The standard plasmid PMD19T-GryAO of *C. pseudotuberculosis* biovar ovis (concentrations of 2.8 × 10^2^ copies/μL, 2.8 × 10^4^ copies/μL, 2.8 × 10^6^ copies/μL) and the standard plasmid PMD19T-GryAE of *C. pseudotuberculosis* biovar equi (concentrations of 2.5 × 10^2^ copies/μL, 2.5 × 10^4^ copies/μL, 2.5 × 10^6^ copies/μL) were used as templates. The established HRM method was applied for intra-assay and inter-assay reproducibility tests. The results (Table 1) showed that the coefficients of variation (CVs) for both the intra-assay and inter-assay were less than 1.0%, indicating good reproducibility of the established HRM method.

### 3.8. Clinical Sample Testing

A total of 133 nasal swabs suspected of CLA were randomly collected. All samples were mixed thoroughly with sterile phosphate-buffered saline (PBS, pH 7.2~7.4) by vigorous shaking to prepare suspensions. After extracting bacterial DNA, the samples were tested using the HRM method established in this study (Table 2). Of these 133 nasal swab samples, 26 tested positive, corresponding to a positive rate of 19.5%. To verify the reliability of these results, the same 133 samples were simultaneously tested using the TaqMan-qPCR method. The detection results were completely consistent with HRM, also identifying 26 positive samples with a positivity rate of 19.5% (26/133), indicating that the HRM method established in this study has excellent detection accuracy. Further analysis of the HRM curve results revealed that two positive samples exhibited significant deviations in melting temperature (Tm values) compared to the remaining 24 positive samples, with values of 86.88 and 86.91, respectively. Based on the classification criteria established in this study (Tm value 86.92 ± 0.05 °C corresponding to *C. pseudotuberculosis* biovar equi), these two positive samples were identified as *C. pseudotuberculosis* biovar equi. To confirm the strain typing, the two positive samples underwent PCR product cloning, sequencing, and sequence alignment analysis. The results showed that their sequences had 100% homology with the published sequences of *C. pseudotuberculosis* biovar equi strains in GenBank. The gyrA gene fragment (140 bp) sequences of the two goat nasal samples with molecular detection positive for *C. pseudotuberculosis* biovar equi have been successfully submitted to the GenBank database, and the obtained accession numbers are (PZ166169) and (PZ166170). Additionally, for the 26 nasal swab samples that confirmed as *C. pseudotuberculosis*-positive had been cultured and identified, bacterial colonies morphologically resembling *C. pseudotuberculosis* were isolated, purified, and identified via 16S rRNA sequencing. A total of four strains of *C. pseudotuberculosis* biovar ovis were isolated, but no *C. pseudotuberculosis* biovar equi strains were successfully isolated. The lack of culture confirmation is a limitation that prevents definitive biovar identification. This result thus only confirms the presence of a biovar equi-like gyrA gene fragment in goat nasal swabs at the molecular level.

## 4. Discussion

*Corynebacterium pseudotuberculosis* is a neglected zoonotic pathogen that not only causes significant economic losses in the livestock industry but also poses a potential threat to public health, particularly for individuals in close contact with infected animals. There are two main biovars of *Corynebacterium pseudotuberculosis*: biovar ovis and biovar equi. Current research suggests that the biovar ovis may have evolved from the biovar equi [32], potentially driven by host adaptation and differentiation. The biovar ovis primarily infects small ruminants such as goats and sheep, while the biovar equi exhibits a broader host range, including horses, camels, cattle, and other animals, and demonstrates a relatively higher mutation rate [33]. Despite the biovar equi’s wider host spectrum, all reported human infection cases to date have been caused by the biovar ovis [7,34]. This divergence in host preference suggests that there may be significant differences in the pathogenic mechanisms between the two biovars. Therefore, establishing accurate, efficient, and rapid identification methods to distinguish these two biovars will not only help clarify the epidemiological characteristics of *C. pseudotuberculosis* but also provide essential tools for in-depth exploration of their infection mechanisms and pathogenic processes.

HRM is a highly efficient and cost-effective tool for mutation screening, widely used for high-throughput genotyping and SNP detection [35]. Comparatively, traditional identification of *C. pseudotuberculosis* relies on bacterial isolation, identification, and nitrate reduction tests, which are cumbersome and time-consuming. Molecular biology methods such as restriction fragment length polymorphism (RFLP) of chromosomal DNA [22], pulsed-field gel electrophoresis (PFGE), BOX-PCR, random amplified polymorphic DNA (RAPD), and amplification of DNA fragments surrounding rare restriction sites (ADSRRS) [33], as well as multiplex PCR [21], can be used for differentiation. However, these methods require subsequent electrophoresis for result verification, a step that is labor-intensive and prone to cross-contamination. The HRM detection method established in this study involves fully closed-tube operations, not only reducing procedural steps but also avoiding aerosol contamination caused by opening tubes. Compared to MALDI Biotyper and the overall genome-relatedness index (OGRI) [20], the HRM-based diagnostic method developed in this study only requires adding the minimally interfering saturated fluorescent dye Syto9 [36] to a standard PCR reaction system. This method based on conserved and validated SNP targets, allows for rapid, cost-effective, and straightforward differentiation between *C. pseudotuberculosis* biovar ovis and *C. pseudotuberculosis* biovar equi using a fluorescence quantitative PCR instrument with HRM analysis capabilities. Additionally, the amplification process is displayed in real-time, making results intuitive and easy to interpret.

Bacterial DNA gyrase catalyzes energy-dependent negative supercoiling of DNA, playing a crucial role in DNA replication, recombination, and transcription. The *gyrA* gene encodes subunit A of DNA gyrase, which is highly conserved and exists as a single copy in all bacteria. Utilizing *gyrA* as the target gene for identification simplifies the process of genetic analysis and comparison [37]. In studies of the *Bacillus* genus, researchers have found that the *gyrA* gene effectively resolves phylogenetic relationships among multiple closely related species within the *Bacillus subtilis* group, significantly outperforming the resolution capability of the 16S rRNA gene [38]. Additionally, since bacterial resistance to quinolone antibiotics is associated with variations in the *gyrA* gene [39], this gene is also applied in molecular detection of quinolone antibiotic resistance. It has been used for resistance screening in important pathogens such as *Salmonella* and *Campylobacter jejuni* [40,41]. While this study focuses on biovar differentiation, future work could explore correlations between *gyrA* mutations and antibiotic resistance in *C. pseudotuberculosis*, simultaneously screening for resistance-related variants and expanding the application scope of the HRM detection method. This will provide potential application value for this method in veterinary clinical practice and public health surveillance.

Currently, research on the *gyrA* gene as a molecular marker for biovar differentiation in *C. pseudotuberculosis* remains relatively limited. This study compared 90 strains of *C. pseudotuberculosis* biovar ovis and 77 strains of *C. pseudotuberculosis* biovar equi published in NCBI. It was found that the *gyrA* gene exists in all published genome sequences of *C. pseudotuberculosis* strains. The nucleotide homology comparison results showed that the FJ-PN strain shares 99.92–99.96% nucleotide homology with the gyrA gene of other *C. pseudotuberculosis* biovar ovis in GenBank, and 99.10–99.18% with *C. pseudotuberculosis* biovar equi, confirming the high conservation of this gene. However, there are also some differences between *C. pseudotuberculosis* biovar ovis and biovar equi, forming distinct evolutionary branches. In this study, analysis of gyrA gene fragments identified four divergent nucleotide positions within the *gyrA* gene fragment (nucleotide positions 2388–2528) between the two biovars. A total of four single nucleotide polymorphisms (SNPs) were detected at positions 2454, 2457, 2495, and 2499 of the *gyrA* gene. The nucleotide residues at these positions were identified as A, T, T, and C in biovar ovis, whereas biovar equi exhibited corresponding substitutions of G, C, C, and T. By optimizing reaction conditions, an HRM detection method targeting this region was established. The results showed that these four mutated bases caused *C. pseudotuberculosis* biovar ovis to form a single melting peak (Tm value) at 86.16 ± 0.03 °C, while *C. pseudotuberculosis* biovar equi formed a single melting peak (Tm value) at 86.92 ± 0.03 °C, with a peak difference of 0.76 ± 0.03 °C. This difference allows clear differentiation between the two biovars. During the comparison, it was also observed that this gene shares higher homology with species in the genus *Corynebacterium*, such as *C. ulcerans* (88.58–89.16%), and *C. diphtheriae* (80.10–80.31%), while nucleic acid homology with other members is below 80.00%. The specificity of primers is crucial for the accuracy of monitoring results. Therefore, when designing primers, interference from *C. ulcerans* and *C. diphtheriae* must be excluded. This study utilized upstream and downstream primers CP-GR2F and CP-GR2R, which have base sequence mismatches with *C. ulcerans* and *C. diphtheriae*. Experimental validation confirmed the primers’ specificity, eliminating interference from the three bacterial species. *C. pseudotuberculosis*, *C. ulcerans*, and *C. diphtheriae* are all pathogenic microorganisms with zoonotic potential, capable of causing abscesses in humans or animals, collectively referred to as the *Corynebacterium diphtheriae* complex [42]. Given the application value of the HRM method in the differential diagnosis of the two biovars of *C. pseudotuberculosis* in this study, future efforts could further optimize the HRM detection method to facilitate epidemiological investigations of pathogens such as *C. pseudotuberculosis*, *C. ulcerans*, and *C. diphtheriae*, which may cause abscesses in animals or humans.

This study established an HRM method for detecting *C. pseudotuberculosis* biovar ovis and *C. pseudotuberculosis* biovar equi, optimized on the LC96 real-time PCR instrument for primer concentration and reaction temperature. Under the optimized conditions, the method demonstrated strong reproducibility, with intra-batch and inter-batch reproducibility both <0.1%, indicating that the melting curves of *C. pseudotuberculosis* biovar ovis and *C. pseudotuberculosis* biovar equi have relatively stable Tm values, allowing differentiation between the two based on Tm value differences. Additionally, under these conditions, the method’s lower detection limits for *C. pseudotuberculosis* biovar ovis and *C. pseudotuberculosis* biovar equi were 28 copies/μL (2.8 × 10^1^ copies/μL) and 25 copies/μL (2.5 × 10^1^ copies/μL), respectively, showing higher sensitivity compared to conventional PCR methods [43]. The HRM sensitivity approached the detection limit of the TaqMan method (10 copies/assay) [31], while this method does not require expensive probes and can detect both *C. pseudotuberculosis* biovars in a single reaction, making it more economical and suitable for large-scale clinical sample screening in resource-limited laboratories. To validate the method’s detection capability in clinical samples, 133 clinical nasal swabs were tested, revealing a positivity rate of 19.5%. Parallel testing with the TaqMan-qPCR method yielded consistent results, demonstrating the method’s accuracy in practical applications. During the analysis of HRM results, Tm value variations were observed in positive samples. Two samples showed melting curve Tm values (86.92 ± 0.05 °C) characteristic of *C. pseudotuberculosis* biovar equi, suggesting possible colonization of this biovar in goats. Although TaqMan-qPCR confirmed these samples as *C. pseudotuberculosis*-positive, it could not differentiate between biovars. Therefore, qPCR products were subjected to gel electrophoresis, fragment recovery, and cloning sequencing. Blast analysis of sequencing results matched *C. pseudotuberculosis* biovar equi sequences in NCBI. The two positive samples showed molecular characteristics consistent with biovar equi based on gyrA SNP analysis and sequencing. Although *C. pseudotuberculosis* biovar equi has a broader host range, to our knowledge, no previous studies have reported the isolation or molecular detection of *C. pseudotuberculosis* biovar equi in goats. Despite molecular detection identifying two biovar equi-positive samples, subsequent bacterial isolation and identification failed to recover corresponding strains. The discrepancy between HRM and bacterial isolation results may stem from multiple factors, such as goats nasal fluid might harbor low *C. pseudotuberculosis* biovar equi load or dormant bacteria, hindering competition with other nasal bacteria. Additionally, the culture methods employed (Section 2.3.3) may have been suboptimal for isolating fastidious organisms like *C. pseudotuberculosis* biovar equi, despite standardized preservation, transport, and culture conditions basically meeting the growth requirements of *C. pseudotuberculosis.* The clinical samples may contain excessive contaminants, a low bacterial load, or be in a dormant state, coupled with unpredictable accidents during transportation, conventional culture methods may fail to revive the bacteria. To mitigate these challenges, future work will explore two strategies: adding potassium tellurite to suppress contaminant growth and enhance the isolation rate of target *C. pseudotuberculosis* [17]; and supplementing media with resuscitation-promoting factor (Rpf) to stimulate the activity of dormant strains [44]. Moreover, HRM targets DNA rather than viable bacteria, allowing detection even if bacteria are dead or non-culturable, which may explain molecular detection success despite culture failure. It should be emphasized that the molecular positive result only indicates the presence of the pathogen’s nucleic acid in the goat nasal cavity, and currently cannot be defined as an established infection due to the lack of viable bacterial isolation; the result only suggests that the identification of samples with equi-like melting profiles and SNPs is based solely on molecular screening and represents preliminary evidence rather than definitive biovar assignment, which needs to be further verified by large-scale sample surveys and optimized culture experiments. In addition, we will continue to monitor newly deposited sequence data to better understand the gyrA gene polymorphisms and refine our assay accordingly in future studies. Among other positive nasal swabs, only four *C. pseudotuberculosis* biovar ovis strains were successfully isolated by culture, whereas HRM detected 24 positive samples, and demonstrated higher efficiency of molecular detection. These findings also indicate that *C. pseudotuberculosis* can be excreted from the body via nasal secretions. Although current research indicates *C. pseudotuberculosis* primarily spreads via skin contact and wounds, with no confirmed aerosol transmission through sneezing or coughing, the detection of this pathogen in goat nasal swabs confirms that lung pus can release *C. pseudotuberculosis* via nasal discharge, potentially forming a transmission route [45], posing exposure risks for high-risk groups like veterinarians, farmers, and slaughterhouse workers. Importantly, nasal swab testing is irreplaceable for controlling visceral CLA. Unlike superficial CLA, which can be preliminarily diagnosed through subcutaneous abscesses, visceral CLA lacks obvious symptoms, with studies indicating predominant pulmonary lesions [46] and higher treatment difficulty and mortality [47]. Therefore, routine nasal swab testing in goats enables early detection of visceral and subclinical cases, facilitating timely isolation, culling, and control measures to reduce CLA transmission risks and economic losses in herds.

## 5. Conclusions

The HRM method established in this study exhibits high sensitivity, excellent specificity, and simple operation, enabling the rapid detection of *C. pseudotuberculosis* in goat nasal swabs and preliminary biovar typing. This method is suitable for large-scale clinical sample screening and offers the advantage of rapid biovar differentiation, thus overcoming the limitations of traditional methods. Additionally, this study indicates that two biovar equi-like gene fragments were detected in goat nasal swabs via molecular screening. Future research should expand sample sources and sizes, optimize bacterial isolation and culture conditions, clarify the epidemiological characteristics and pathogenicity of the equi biovar in goat herds, and provide more comprehensive evidence for the precise prevention and control of CLA.

## Figures and Tables

**Table 1:** Reproducibility analysis of the HRM method.

Concentration of Plasmid Standards (Copies/μL)	Intra-Assay		Inter-Assay		Intra-Assay		Inter-Assay	
	Tm1 X ± SD	CV/%	Tm1 X ± SD	CV/%	Tm2 X ± SD	CV/%	Tm2 X ± SD	CV/%
N1/N2 × 10^6^	86.16 ± 0.011	0.013	86.15 ± 0.028	0.024	86.92 ± 0.015	0.017	86.92 ± 0.021	0.024
N1/N2 × 10^4^	86.16 ± 0.017	0.24	86.15 ± 0.03	0.034	86.92 ± 0.02	0.01	86.91 ± 0.027	0.03
N1/N2 × 10^2^	86.15 ± 0.02	0.023	86.16 ± 0.04	0.046	86.90 ± 0.02	0.023	86.91 ± 0.038	0.044

Note: N1 = 2.8 (*C. pseudotuberculosis* biovar ovis), N2 = 2.5 (*C. pseudotuberculosis* biovar equi).

**Table 2:** Detection results of clinical goat nasal swab samples by HRM and TaqMan-qPCR methods.

	Nasal Swab		
Detection Method	Positive Number	Negative Number	Positivity Rate
HRM	26	107	19.5%
Taqman-qPCR	26	107	19.5%

## Data Availability

The original contributions presented in this study are included in the article/Supplementary Materials. Further inquiries can be directed to the corresponding author.

## Supplementary Materials

The following supporting information can be downloaded at: https://www.mdpi.com/article/10.3390/vetsci13040372/s1, Table S1: Strain SNP Difference Table; Table S2: Information of Bacterial Strains Employed in Phylogenetic Tree Construction and Multiple Sequence Alignment.
