# Supplementary material for: Development and Validation of a High-Resolution Melting (HRM) Method for Differentiating Ovis and Equi Biovars of Corynebacterium pseudotuberculosis
Source: Vet Sci. 2026 Apr 13;13(4):372. doi: 10.3390/vetsci13040372 (PMC13120198; doi:10.3390/vetsci13040372)
Supplement: Supplementary file 1 [file vetsci-13-00372-s001.zip › vetsci-4211294-supplementary.pdf]

Table S1. Strain SNP Difference Table

GenBank NO.	Biovar	Distinguish conserved SNP sites of biological types (target fragment locations)			
		**SNP1(2454)**	**SNP2(2457)**	**SNP3(2495)**	**SNP4(2499)**
CP011474	ovis	A	T	T	C
CP016826	ovis	A	T	T	C
CP010889	ovis	A	T	T	C
CP003152	ovis	A	T	T	C
CP052882	ovis	A	T	T	C
CP024995	ovis	A	T	T	C
CP013698	ovis	A	T	T	C
CP003385	ovis	A	T	T	C
CP074370	ovis	A	T	T	C
CP002251	ovis	A	T	T	C
CP013146	ovis	A	T	T	C
CP046641	ovis	A	T	T	C
CP003062	ovis	A	T	T	C
CP008922	ovis	A	T	T	C
CP013699	ovis	A	T	T	C
CP017291	equi	G	C	C	T
CP085676	equi	G	C	C	T
CP003540	equi	G	C	C	T
CP003061	equi	G	C	C	T
CP015192	equi	G	C	C	T
CP121343	equi	G	C	C	T
CP012136	equi	G	C	C	T
CP017384	equi	G	C	C	T
CP115164	equi	G	C	C	T
CP115163	equi	G	C	C	T
CP003652	equi	G	C	C	T

Table S2. Information of Bacterial Strains Employed in Phylogenetic Tree Construction and Multiple Sequence Alignment

GenBank NO.	strain	Organism	Host	Area	Collection date
CP011474	12C	Corynebacterium pseudotuberculosis biovar ovis	Ovis aries	Brazil	2009
CP016826	MEX29	Corynebacterium	Ovis aries	Mexico	2013

GenBank NO.	strain	Organism	Host	Area	Collection date
		pseudotuberculosis biovar ovis			
CP010889	226	Corynebacterium pseudotuberculosis biovar ovis	Ovis aries	USA	2007
CP003152	339-5	Corynebacterium pseudotuberculosis biovar ovis	Ovis aries	UK	1999
CP052882	IJ-2	Corynebacterium pseudotuberculosis biovar ovis	Capra hircus	South Korea	2019
CP024995	Km01	Corynebacterium pseudotuberculosis biovar ovis	Capra hircus	China	2017
CP013698	PO2224-1	Corynebacterium pseudotuberculosis biovar ovis	Capra hircus	Portugal	2015
CP003385	P54B96	Corynebacterium pseudotuberculosis biovar ovis	Connachaetes taurinus	South Africa	2012
CP074370	12CS0282	Corynebacterium pseudotuberculosis biovar ovis	Capra hircus	Germany	2021
CP002251	I 19	Corynebacterium pseudotuberculosis biovar ovis	Bos taurus	Israel	2019
CP013146	N1	Corynebacterium pseudotuberculosis biovar ovis	Ovis aries	Equatorial Guinea	2014
CP046641	PAT16	Corynebacterium pseudotuberculosis biovar ovis	Ovis aries	Argentina	2007
CP003062	4202-A	Corynebacterium pseudotuberculosis biovar ovis	Ovis aries	Australia	2011
CP008922	48252	Corynebacterium pseudotuberculosis biovar ovis	Capra hircus	Norway	2014
CP013699	E56	Corynebacterium pseudotuberculosis biovar ovis	Ovis aries	Egypt	2015
CP017291	MEX30	Corynebacterium pseudotuberculosis biovar equi	Equns caballus	Mexico	2013
CP085676	MB45	Corynebacterium pseudotuberculosis biovar equi	Equns caballus	USA	2001
CP003540	258	Corynebacterium pseudotuberculosis biovar equi	Equns caballus	Belgium	2019
CP003061	CIP 5297	Corynebacterium	Equns caballus	Kenya	2011

GenBank NO.	strain	Organism	Host	Area	Collection date
		pseudotuberculosis biovar equi			
CP015192	34	Corynebacterium pseudotuberculosis biovar equi	Bubalus bubalis	Egypt	2016
CP121343	BA3	Corynebacterium pseudotuberculosis biovar equi	Vicugna pacos	China	2023
CP012136	E19	Corynebacterium pseudotuberculosis biovar equi	Equns caballus	Chile	2015
CP017384	I37	Corynebacterium pseudotuberculosis biovar equi	Bos taurus	Israel	2016
CP115164	CVUAS 55832	Corynebacterium pseudotuberculosis biovar equi	Camelus dromedarius	Germany	2015
CP115163	CVUAS 32689	Corynebacterium pseudotuberculosis biovar equi	Vicugna pacos	Germany	2020
CP003652	Cp162	Corynebacterium pseudotuberculosis biovar equi	Camelus	UK	2019
CP002790	809	Corynebacterium ulcerans	Homo sapiens	Brazil	2011
CP011913	FRC58	Corynebacterium ulcerans	Homo sapiens	France	2010
CP002791	BR-AD22	Corynebacterium ulcerans	Canis lupus familiaris	Brazil	2011
AP019663	FH2016-1	Corynebacterium ulcerans	Homo sapiens	Japan	2016
CP103876	263322	Corynebacterium diphtheriae	Homo sapiens	Poland	2022
CP047201	DSM 44123	Corynebacterium diphtheriae	Homo sapiens	USA	2020
CP025209	ISS3319	Corynebacterium diphtheriae	Homo sapiens	Italy	1997
CP040523	CD1032	Corynebacterium diphtheriae	Homo sapiens	Bangladesh	2017
CP134941	MoG225L22A	Mesomycoplasma ovipneumoniae	Capra hircus	Austria	2022
CP079200	150	Mesomycoplasma ovipneumoniae	Ovis aries	Bosnia and Herzegovina	2009
CP065579	G1313-94	Mycoplasma mycoides subsp. capri	Ammotragus lervia	Germany	1994
CP017125	M1601	Mycoplasma capricolum subsp. capripneumoniae	Capra hircus	China	2013
